# Cofactor-enhanced food allergy to presumed soy storage proteins in a pediatric patient

**DOI:** 10.31744/einstein_journal/2025RC1044

**Published:** 2025-02-07

**Authors:** Ana Raquel Pinto, Fabrícia Carolino

**Affiliations:** 1 Centro Hospitalar Universitário de Santo António Allergy and Clinical Immunology Department Porto Portugal Allergy and Clinical Immunology Department, Centro Hospitalar Universitário de Santo António, Porto, Portugal.

**Keywords:** Anaphylaxis, Ibuprofen, Cofactor, Food allergy, Anti-inflammatory agents, non-steroidal, Child, Soy proteins, Food hypersensitivity

## Abstract

Food allergies are the leading cause of anaphylaxis in children. Cofactors, such as exercise and non-steroidal anti-inflammatory drugs, may influence the occurrence and severity of allergic reactions to food. However, despite their relevance, the underlying mechanisms of cofactor-enhanced food allergies remain poorly understood. We report the case of a 12-year-old girl with mitochondrial DNA depletion syndrome who was referred to an allergy appointment due to suspected ibuprofen hypersensitivity. Detailed anamnesis, laboratory assessment, and negative drug challenge results excluded this diagnosis; however, continued follow-up revealed a crucial pattern on patient reactions that led to the diagnosis of a cofactor-enhanced food allergy with the presumed involvement of soy storage proteins and non-steroidal anti-inflammatory drugs. Our purpose was to highlight the non-negligible role of cofactors in food allergies and the importance of early identification. Moreover, to underscore the relevance of maintaining patient follow-up, as new information may arise and redirect the diagnosis.

## INTRODUCTION

Food allergies are the leading cause of anaphylaxis in the pediatric population and, according to some studies, in adults.^(1)^ Numerous food sources have demonstrated the potential to elicit allergic reactions depending on the individual's susceptibility, threshold dose required, and type of allergen.^(1,2)^ Soy, a major food allergy source in Europe, has an overall self-reported physician-diagnosed allergy prevalence of 0.3% which is generally higher in children, although data regarding the adult population are sparse.^(3)^ Other common food allergies in Europe include cow's milk, eggs, wheat, peanuts, tree nuts, fish, and shellfish.^(3)^

The severity of an allergic reaction is often unpredictable because the same individual may develop symptoms of varying degrees of severity with the same food source as a trigger. Cofactors may contribute to this heterogeneity, influencing reaction occurrence/severity; however, the underlying mechanisms are still not fully understood.^(4)^ According to the literature, cofactors play a more prominent role in adults than in pediatric patients (30% *versus* 14–18.3%).^(4)^ The most commonly involved are non-steroidal anti-inflammatory drugs (NSAIDs) and exercise, followed by alcohol intake. However, the roles of exercise and infection appear to be more relevant in pediatric patients.^(1,2,4)^

Several terms have been applied to these reactions since their first description, including food-dependent exercise-induced anaphylaxis (FDEIA) or wheat-dependent exercise-induced anaphylaxis. However, the currently accepted, more generalist designation is cofactor-enhanced food allergy (CEFA). Omega-5-gliadin (a wheat storage protein) and non-specific lipid transfer proteins (nsLTPs) are the most frequently associated food allergens in the Mediterranean region.^(5)^

We report a pediatric case initially assessed as a suspected drug allergy, which turned out to be a CEFA, with the presumed involvement of soy storage proteins and NSAID intake.

## CASE REPORT

A 12-year-old girl with a history of mitochondrial DNA depletion syndrome and consequent psychomotor developmental delay, who was fed through percutaneous endoscopic gastrostomy (PEG), was referred to our allergy department for suspected ibuprofen hypersensitivity.

Her caregiver reported two previous reproducible episodes of palpebral edema with conjunctival hyperemia and pruritus 30 min after ibuprofen intake via PEG (100 mg, ~6 mg/kg). The symptoms subsided following oral prednisolone administration. Previous exposure to ibuprofen and subsequent tolerance to paracetamol was confirmed. Additionally, she had a history of walnut-induced anaphylaxis and developed facial angioedema and bronchospasm 20 min after consuming a homemade walnut muffin; multiple episodes of facial angioedema immediately after *Rosaceae* fruit ingestion were also reported. Symptoms associated with other foods, such as nuts, peanuts, or milk, were excluded. The child's caregiver was provided with avoidance measures (NSAIDs, tree nuts, peanuts, and *Rosaceae* fruits) and an emergency treatment plan (epinephrine autoinjector and oral antihistamines/corticosteroids).

An *in vitro* study was initially preferred for food-related symptoms because of patient comorbidities. The results showed increased total serum immunoglobulin E (IgE 524 kUA/L) and IgE-mediated sensitization to walnuts (specific IgE 3.69 kUA/L). To better clarify the patient's symptoms with *Rosaceae* fruits, an ImmunoCAP ISAC assay was performed, revealing primary sensitization to nsLTPs (Jug r 3 = 9.8 ISU-E, Cor a 8 = 3.3 ISU-E, Ara h 9 = 6 ISU-E, Pru p 3 = 4.1 ISU-E, Tri a 14 = 0.5 ISU-E), food-specific storage proteins (Gly m 5 = 5.1 ISU-E, Gly m 6 = 4.4 ISU-E, Ara h 3 = 1.2 ISU-E, Ana o 2 = 0.7 ISU-E, Ses i 1 = 0.7 ISU-E), and serum albumin (Bos d 6 = 1.2 ISU-E) (Table 1).

**Table 1:** Summary results of *in vitro* testing

Total IgE				
524 (kUA/L)				
**Specific IgE**				
	Walnut	3.69 (kUA/L)	
**ImmunoCAP ISAC**				
Species-specific food components				
Soy		Gly m 5†	5.1 (ISU-E)
		Gly m 6†	4.4 (ISU-E)
Peanut		Ara h 3†	1.2 (ISU-E)
Cashew		Ana o 2†	0.7 (ISU-E)
Sesame		Ses i 1†	0.7 (ISU-E)
Cross-reactive components				
Walnut		Jug r 3‡	9.8 (ISU-E)
Hazelnut		Cor a 8‡	3.3 (ISU-E)
Peanut		Ara h 9‡	6 (ISU-E)
Peach		Pru p 3‡	4.1 (ISU-E)
Wheat		Tri a 14‡	0.5 (ISU-E)
Cow's milk/meat		Bos d 6§	1.2 (ISU-E)

† Storage proteins;

‡ non-specific lipid transfer proteins;

§ serum albumin.

Summary results of total IgE, specific IgE, and ImmunoCAP ISAC, divided by species-specific food and cross-reactive components.

A drug challenge with ibuprofen (150 mg, ~8 mg/kg) was negative; however, subsequent drug exposure was inconsistently tolerated. The child's caregiver was instructed to track reactions, and a subsequent review of the records unveiled that a reaction to ibuprofen only occurred when its intake was associated with the simultaneous ingestion of a milk-based soy-containing medical nutritional supplement (via PEG), which she tolerated alone. Considering the patient's comorbidities, clinical complications, and the absence of safe and validated protocols, an oral co-challenge with this supplement and ibuprofen was not performed. Consequently, a diagnosis of CEFA involving soy storage proteins and NSAIDs was presumed. The parents received recommendations regarding the avoidance of the combined use of soy-containing products and NSAIDs, along with the avoidance of tree nuts, peanuts, and *Rosaceae* fruits.

This study was approved by the Research Ethics Committee of *Unidade Local de Saúde Santo António*, 2024.186 (CC-CAC/159-CE).

## DISCUSSION

The initial approach to a suspected food allergy requires thorough anamnesis, followed by *in vivo* and/or *in vitro* testing according to the suspected allergen and the nature of the reaction. In the case of a negative result, despite a suggestive anamnesis, the possible involvement of cofactors should be considered. Performing oral food challenges combined with cofactors may be helpful; however, standardized and widely accepted challenge protocols are lacking, thereby reducing their clinical validity.^(4,6)^

The involvement of soy storage proteins in CEFA has rarely been reported in the literature and is mostly associated with FDEIA. The mechanism of action of NSAIDs as cofactors is not fully understood but appears to be both dependent and independent of cyclooxygenase inhibition. Two main hypotheses have been suggested: 1) an alteration of intestinal permeability leading to increased allergen absorption, and 2) a direct effect on basophils and mast cells.^(1)^

Cofactor-enhanced food allergy management requires avoidance measures, particularly the combination of food allergens and cofactors, and an emergency treatment plan that may occasionally include an epinephrine autoinjector. Although avoidance strategies may seem straightforward, ubiquitous allergens/pan-allergens present a substantial challenge. Moreover, considering that cofactors are common in everyday situations, their full avoidance is difficult. Therefore, patients benefit from individualized dietary counseling.^(1,6)^

Until recently, avoidance measures and emergency medications for accidental exposure were the only options available for managing food allergies; however, different options for oral-specific immunotherapy have emerged, including commercially available products, and new administration routes are under investigation. Much remains unclear regarding their sustained efficacy or whether this treatment can effectively control cofactors or prevent new sensitizations; thus, further studies are required.^(7,8)^

## CONCLUSION

We aimed to highlight the non-negligible role of cofactors in food allergies, which should motivate attempts to identify them during the initial diagnostic workup or following a negative allergy study despite a suggestive anamnesis. The relevance of maintaining patient follow-up after a negative challenge as new information may arise and redirect diagnosis is also evidenced. To the best of our knowledge, this is the first report of a cofactor-enhanced food allergy to the soy storage protein components Gly m 5/6, with the involvement of an non-steroidal anti-inflammatory drug as a cofactor. Further studies are needed to corroborate and reinforce our observations.
